# Pathology skills lab: use of macroscopic tumor models in pathology teaching

**DOI:** 10.1186/s12909-024-05575-z

**Published:** 2024-05-30

**Authors:** Marit Bernhardt, Christine Sanders, Oliver Hommerding, Dora Nagy, Tobias Kreft, Xiaolin Zhou, Glen Kristiansen

**Affiliations:** Institute of Pathology, University Hospital Bonn, Venusberg-Campus 1, Bonn, 53127 Germany

**Keywords:** Simulation, Quality education, Pathology, Pathologist shortage

## Abstract

**Background:**

The shortage of pathologists in Germany, coupled with an aging workforce, requires innovative approaches to attract medical students to the field. Medical education must address different learning styles to ensure that all students are successful.

**Methods:**

The pilot project “Practical Pathology” aims to enhance students' understanding of pathology by providing hands-on experience in macroscopic gross analysis through the use of tumor dummies built from scratch.

**Results:**

An evaluation survey, completed by 63 participating students provided positive feedback on the course methodology, its relevance to understanding the pathology workflow, and its improvement over traditional teaching methods. The majority of students recognized the importance of hands-on training in medical education. Students with previous work experience rated the impact of the course on knowledge acquisition even more positively.

**Conclusion:**

The course improved students' understanding of pathological processes and potential sources of clinical-pathological misunderstanding. An increase in motivation for a potential career in the field of pathology was observed in a minority of students, although this exceeded the percentage of pathologists in the total medical workforce.

## Introduction

Despite the increase in the number of medical students in Germany, the number of pathologists is not increasing at a comparable rate [1, 2]. Instead, the number of practicing pathologists is expected to decrease due to demographic changes. By 2022, approximately 60% of practicing pathologists were 50 years of age and older [2]. Studies have shown that medical students often overlook a career in pathology due to a lack of interest and understanding of the subject matter [3]. Medical education offers the opportunity to inspire future generations of medical students for the subject of pathology in all its variety.

The National Competency-Based Learning Objectives Catalogue for Medicine (NKLM) is a federal German document that defines educational objectives for medical schools throughout the country. It serves as the basis for an upcoming reform of German medical education, known as the Medical Education Master Plan 2020, which aims to comprehensively overhaul and redesign medical education. One of the central goals of the reform is to ensure competency-based medical education with an emphasis on early practice orientation [4–6].

In this context, the Central German Institute for Medical and Pharmaceutical Examinations (“Institut für Medizinische und Pharmazeutische Prüfungsfragen”, IMPP), which is responsible for the preparation of exam questions for state examinations in medical schools, has developed a competence-objected subject catalog (“Gegenstandskatalog”, GK). It summarizes the content that can be included in examinations or state exams. For the subject of anatomical and surgical pathology, knowledge of macroscopic and microscopic changes in the context of inflammatory and neoplastic diseases is required [7].

Traditional pathology education typically involves both a macroscopy and microscopy course, where students examine specimens and analyze changes that, taken together, contribute to a diagnosis. Beyond that, a practical orientation is often not fully feasible. [7–9] However, the daily work of a pathologist is much more than working with a microscope. It includes performing autopsies and participating in interdisciplinary case conferences, molecular analyses, and macroscopy, in which relevant areas of the specimens are selected for further microscopic examination after gross evaluation [10].

Involving students in the dissection of real surgical specimens for educational purposes is debatable. First, correct grossing is essential for diagnosis and must not be compromised by inexperienced medical students, however eager they are. Furthermore, in order to ensure optimal sections for histology, both internal and external standards must be adhered to for routine specimen sampling [11]. Second, formaldehyde, which is unavoidable, has now been classified as a carcinogen and unnecessary exposure should be avoided [12]. Third, there is a low, but still not negative, risk of injury and subsequent infection when using “real” specimens [13]. Fourth, the acquisition of similar specimens from routine samples for large groups of students is difficult, especially if turnaround times are being affected. Although remainder of specimens that have already been signed out may be used after flushing with water to minimize formaldehyde exposure, still a laboratory with fume hoods must be provided as a work station. In addition, programs may not have access to patient specimens for this purpose and providing comparable material to all students may pose an additional challenge. In summary, the use of routine specimens for teaching purposes of medical students is unadvisable and it is important to consider alternative methods of macroscopy education.

In order to meet the requirements of the NKLM and to familiarize students with the profession of pathologists, the Department of Pathology at the University Hospital Bonn has introduced the course "Practical Pathology" as a pilot project in the winter semester 2022/2023. This course aims to make the daily work of pathologists literally "understandable" by adding some virtual flesh to the dry theory.

## Material and methods

### Construction of tumor models

**Fig. 1:**
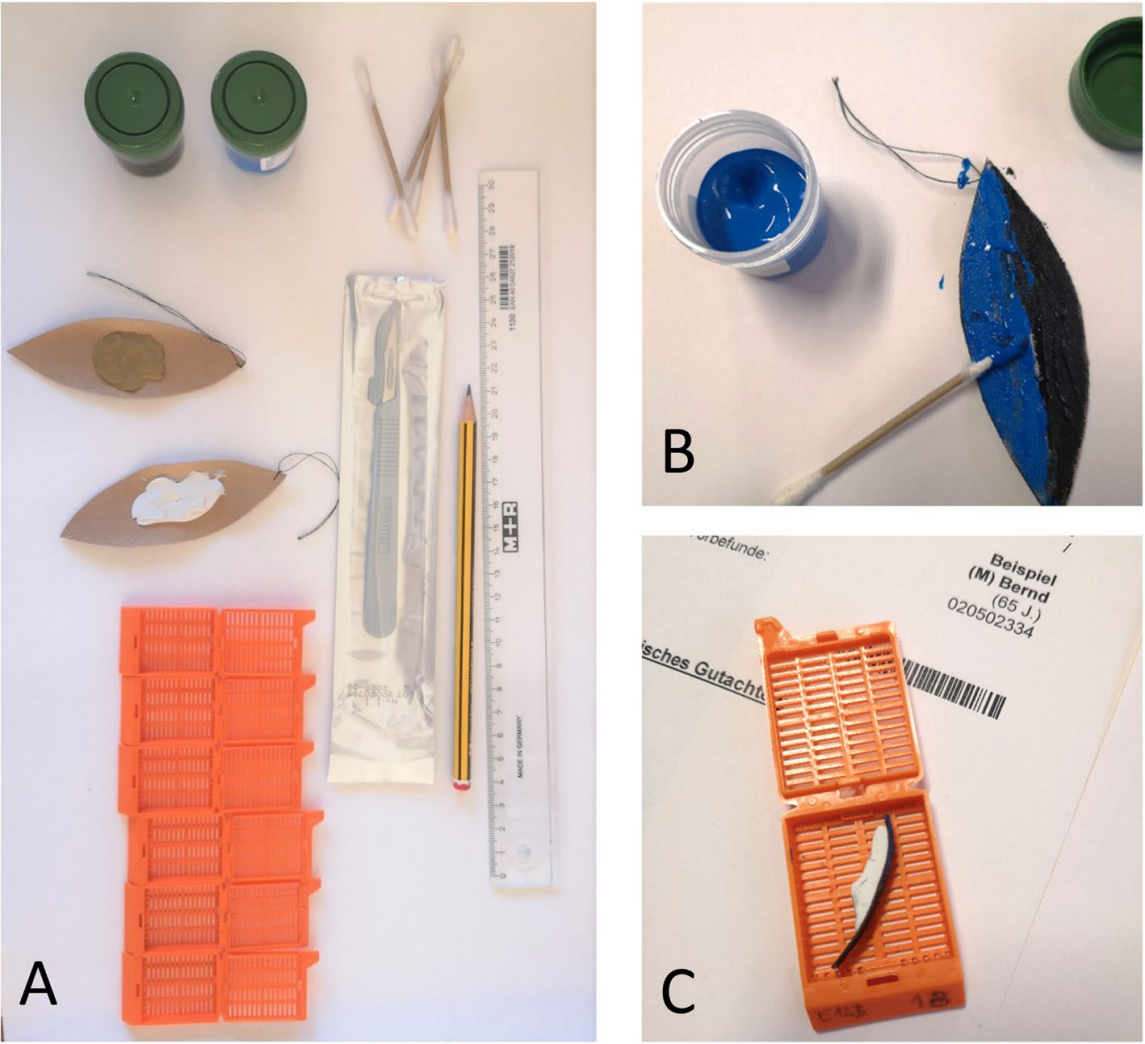
Course material: tumor specimen model, cassettes, ink, ruler and pencil (**A**). Specimen after inking **B** and cutting **C** with report form seen behind

For the "Practical Pathology" course, we created tumor models of a skin spindle excision with squamous cell carcinoma. The skin substitute used was brown artificial leather molded into the shape of a spindle. A cotton thread was attached to the tip of the spindle, marking 12 o'clock. A tumor was created by applying acrylic sealant commercially available at a local hardware store (acrylate sealant Racofix Acrylic White, 21,719, Sopro Bauchemie GmbH, Wiesbaden, Germany). The design of the model took into account the feasibility of using marking ink on it and the ease of producing tumor models quickly, easily and at low cost (Fig. 1).

### Use of tumor models in teaching

Students worked in pairs and each received a tumor model. In addition to the tumor models, students received a pathology requisition form designed for the course with clinical information. Cotton swabs and two different tempera paints were provided for inking the resection margins. Each pair of students was given a ruler, scalpel, and histocassettes to submit their sections. A pathology report mask for recording clinical details and macroscopic descriptions was distributed together with the tumor models (Fig. 1). A lecturer from the Institute of Pathology was always present to answer questions. After a group discussion on macrodissection methods and objectives, students performed dissections in pairs. The group then reviewed their results and discussed potential problems, such as tangential sectioning of small particles or improper visualization of resection margins.

**Fig. 2:**
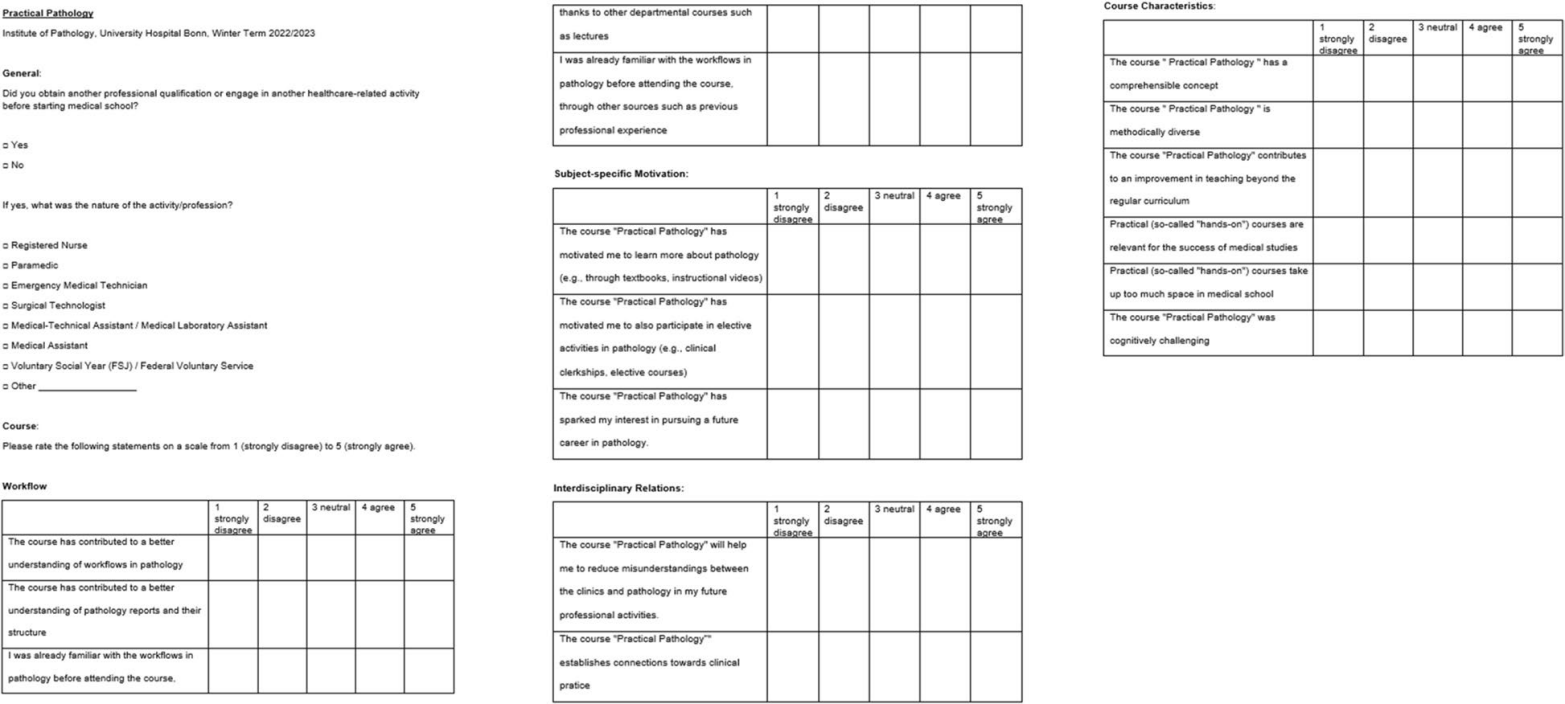
Evaluation sheet handed out to student to document their experiences

Students completed an anonymous and voluntary evaluation survey that inquired about their pre-medical work experience, learning outcomes in understanding pathology workflow, and their motivation to continue to study pathology as a subject (Fig. 2). The survey also assessed students’ perceptions of the course's variety of techniques and its relevance to future interdisciplinary work. Agreement or disagreement was rated on a five-point scale, with 1 representing a low level of agreement ("strongly disagree") and 5 representing a high level of agreement ("strongly agree").

### Statistics

Statistical analysis was performed using IBM SPSS Statistics 27 software (IBM, New York, USA). Data were presented descriptively as absolute and percentages. In addition, means and ranges were determined. The Mann–Whitney U test and the Wilcoxon rank-sum test were used to compare groups. Significance was defined as a *p*-value of < = 0.05, and multiple testing was adjusted to the α-level using the Bonferroni correction.

## Results

**Fig. 3:**
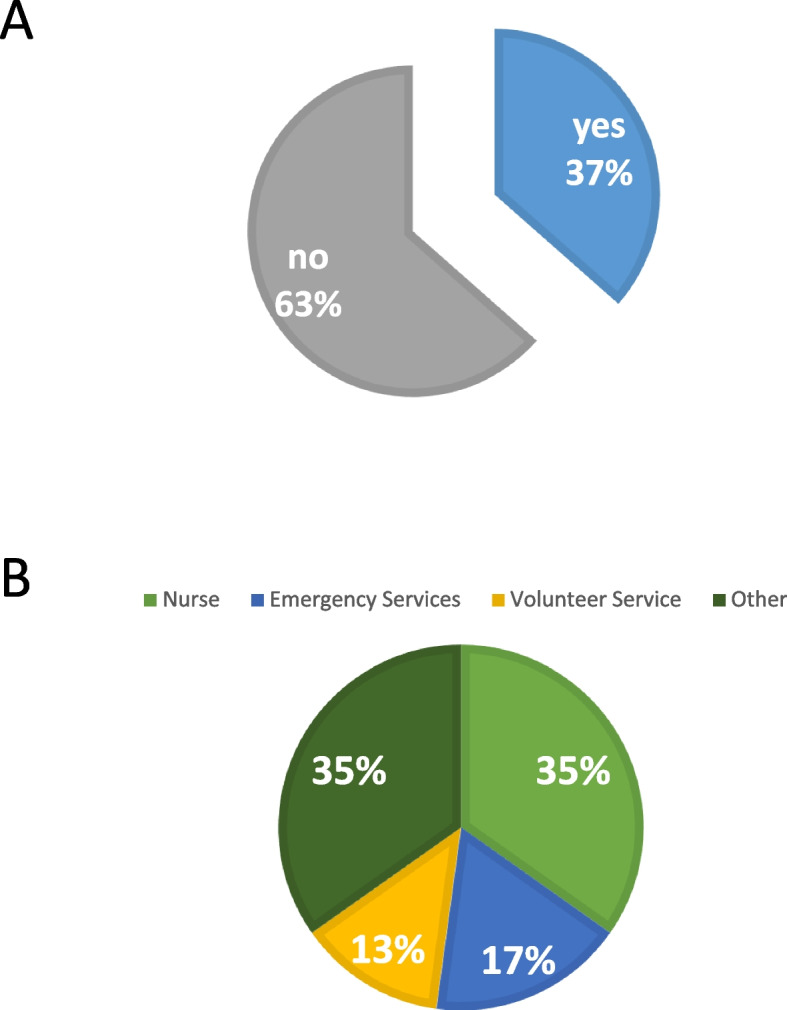
Percentage of students that had been working prior to entering medical school **A** and distribution among professions /services **B**

Of all students enrolled in their fifth semester (*n* = 139), a total of 63 completed the evaluation form. Of these, 23 (36.5%) had previous work or volunteer experience prior to their studies. Professional work experience, if any, was mainly in nursing (12.7%) or emergency services (6.4%). 4.8% of the participants had done a volunteer service. The remainder, 12.7%, included both medical (physiotherapist, medical assistant/physician assistant) and non-medical (mechatronics technician, chemical technician) jobs, as shown in Fig. 3. Overall, none of the students had previous experience in histopathology i.e. from work experience as histotechnician.

Overall, the course received positive evaluations. Students perceived it as an improvement over traditional teaching (mean 3.47; SD 0.953) and appreciated its methodological diversity (mean 3.61; SD 0.894). They also recognized the importance of practical “hands on” courses for the success of medical education (mean 4.32; SD 0.964). Regarding the acquisition of knowledge about the daily work of pathologists, most participants reported that the course gave them a better understanding of the workflow (mean 3.60; SD 0.815). According to the students, information about the workflow was not taught in other classes (mean 2.14; SD 0.948) or known from professional activities before entering medical school (mean 1.75; SD 0.933).

Interestingly, students who endorsed previous employment had significantly more positive ratings regarding knowledge acquisition from the course than those without prior job exposure (mean 4.00 vs. 3.38, *p* = 0.016). Regarding prior knowledge of pathology workflow, previous work experience had no significant effect (*p* > 0.999 in each case) (refer to Table 1).

**Table 1:** Comparison of assessments between students with (*n* = 23, left) and without (*n* = 40, right) work experience prior to the start of medical school; * significant values; *WE work experience*

	Mean WE	Mean no WE	*p*
The course has contributed to a better understanding of workflows in pathology	4.00	3.38	0.016*
The course has contributed to a better understanding of pathology reports and their structure	3.30	3.15	> 0.999
I was already familiar with the workflows in pathology before attending the course, thanks to other departmental courses such as lectures	2.09	2.17	> 0.999
I was already familiar with the workflows in pathology before attending the course, through other sources such as previous professional experience	1.83	1.70	> 0.999

The implementation of the practical pathology course did not lead to an increase in motivation in the field of pathology. The majority of participating students were neutral regarding whether the course played a significant role in inspiring self-motivation for in-depth study (2.92; SD 0.955) or for enrolling in pathology courses in the required elective area (2.83; SD 0.853). In general, the motivation of students to pursue a career in pathology was largely unaffected (2.38; SD 0.923). However, 3.2% of respondents reported an increase or significant increase in motivation in response to this question. The results did not differ significantly whether or not the students had work experience prior to medical school (in-depth study mean 2.85 vs. 3.04 *p* = 0.291, elective mean 2.80 vs. 2.87 *p* = 0.501, career mean 2.48 vs. 2.22 *p* = 0.529).

The teaching of interdisciplinary relationships was positively evaluated. In addition to making clinical connections (3.70; SD 0.994), the course's potential to prevent misunderstandings (3.46; SD 0.997) between clinical staff and pathologists was considered relevant.

## Discussion

In the traditional curriculum of medical education, pathology plays a central role as a didactic core discipline in the advanced / second part of medical education following pre-medical courses. This reflects the way we, as pathologists, see ourselves, in medicine, where diseases are defined and classified primarily in terms of (pathological) anatomy and pathophysiology. Despite this, surveys show that only less than half of medical students are aware of the actual day-to-day work in clinical pathology. In particular, the amount of autopsy work is often overestimated, which may be due to the traditional but outdated image in the media [14]. As a corrective measure, both the German Society of Pathology (DGP) and the Professional Association of German Pathologists (BDP) now provide online information about the profession [10, 15].

Medical school education provides an excellent opportunity to expose future health care professionals to the content and day-to-day practice of the specialty. This is critical, as one of the most commonly cited reasons in surveys for not choosing pathology as a specialty is lack of exposure and knowledge of the subject matter among medical students [3]. Factors that influence specialty choice among current students include interest in the field, intellectual challenge, work-life balance, career opportunities, professional standards, workplace atmosphere, and workplace prestige [14, 16]. These are factors that generally include pathology as a specialty. However, regarding the specific question of preferred specialties, it is evident that the inclination toward internal medicine, surgery, general medicine, and psychiatry is often present before medical school and remains largely unchanged throughout medical school [17]. Inspiring undecided students to consider a career path is also a potential goal of teaching. The presented course concept was evaluated and showed that 3% of the students reported being inspired to pursue a career in pathology as a result of their participation. Considering that pathologists represent only 0.5% of all physicians in Germany, this is an encouraging result [2].

Exposure to the clinical environment or clinical practice is often claimed to be important in the choice of specialty [18]. In addition, students with premedical experience in health sciences tend to perform better in medical school [19]. In our cohort, previous work experience did not appear to influence motivation for pathology education or residency. However, motivation for pathology was not significantly lower in this group either.

In didactics, we often speak of different learning types and, more recently, of different learning styles. A common concept in medical didactics is the "VARK" model—visual, auditory, reading/writing, and kinaesthetic—which identifies four distinct learning styles. These are the visual style, in which content is conveyed using pictures, graphics, etc.; the auditory style, in which knowledge is conveyed through lectures and/or discussions; the reading/writing style, in which teaching and learning is text-based; and the kinaesthetic style, in which knowledge is acquired through direct practical application [20]. While it was initially thought that most people preferred a single learning style, it has now been shown that most people use a variety of learning styles [21, 22]. In addition, different teaching methods have been associated with different efficacy. For example, a lecture is associated with an efficacy of 5%, meaning that students will remember 5% of the content presented. In contrast, learning by performing a task is said to achieve efficacies of up to 75% [23]. The required competence orientation of medical education, which the NKLM already bears in its name, refers to the skills, abilities and professional attitudes of future physicians. The three levels of competence are factual knowledge, action and reasoning knowledge, and action competence. The ability to explain and classify terms and concepts falls under the category of action and reasoning knowledge, while action competence pertains to the ability to carry out activities independently or under supervision.

The teaching of pathology currently meets the criteria for multimodal didactics and the NKLM in several aspects [24]. Microscopy teaching incorporates auditory, kinaesthetic, and visual elements, allowing students to complement their factual knowledge with practical knowledge and skills. The digitization of histological specimens also provides the opportunity for self-directed learning through online platforms independent of traditional coursework [8, 9]. However, simulation-based learning, as is common practice in other disciplines, for example with resuscitation training, suturing courses or other widely available so-called skills labs, has, to our knowledge, not been routinely established in pathology [6, 25, 26]. Skills labs similar to ours, such as grossing of a salivary gland model presented by Alcaraz et al*.* are unique and rare pilot projects [27]. This is particularly unfortunate because the use of simulation-based learning in teaching would be beneficial for strengthening practical skills. A wide range of learning styles can be accommodated, possibly more than in a case-based seminar or a lecture alone [27, 28]. Students’ evaluations seem to agree, as they consider the use of practice-based courses relevant to their academic success. With regard to the integration of new teaching and learning methods, it is important that existing resources are not further strained. With a material cost of about 10 euro cents and a production time of about 1 min, the models adopted by our department offer a cost-effective and time-efficient solution. This makes them highly suitable for long-term implementation with minimal impact on the teaching budget. In addition, the tumor models presented can be used in any standard lecture hall as they are made from dry materials and do not drop or vaporize preservatives. Alternative options used i.e. in suturing or fine needle aspiration skills labs such are animal specimens or fruit [29, 30]. Although especially animal specimens are closest to human material in terms of texture, they need to be purchased freshly before every course, while models like ours may be prepared in larger scale at the beginning of every new semester.

In addition to increasing students' enthusiasm for pathology, the course aimed to improve their understanding of the laboratory workflow from specimen receipt to report release. Previous studies have shown that up to one-third of clinical colleagues does not understand or does not fully understand the information contained in the report [31]. In addition, up to 15% of submitted specimens lack clinical information beyond patient biographical data [32]. This may be taken as a hidden compliment, but it clearly overestimates the knowledge of the pathologist. It includes the assumption that the pathologist, as the “doctor’s doctor”, is endowed with a supernatural sense that renders detailed clinical information superfluous – which has long been scientifically refuted [33]. The most commonly cited justification for the lack of clinical information is the concern that too much information might interfere with an unbiased diagnosis [34]. Therefore, the course not only focused on macroscopic processing, but also emphasized the importance of including relevant information on the submission form to aid in the reporting of findings. The course was successful in positively influencing students' understanding of potential clinical-pathologic misunderstandings. However, it remains to be determined whether this effect will continue to benefit their future clinical work, making it a possible topic for further study.

## Conclusion

The multimodal design of medical education has been associated with improved learning outcomes long before the introduction of the NKLM and problem-based learning. The implementation of a simulation grossing course is positively evaluated by students as it improves the quality of teaching and minimizes misunderstandings between pathologists and clinicians. In addition, the course provides an opportunity to explain students the critical role pathology plays in patient care and to inspire them to consider pathology as a career option, thus mitigating the shortage of future pathologists.

## Data Availability

The datasets used and analysed during the current study are available from the corresponding author on reasonable request.

## Abbreviations

BDP: Bundesverband Deutscher Pathologen / Professional Association of German Pathologists
DGP: Deutsche Gesellschaft für Pathologie / German Society of Pathology
GK: Gegenstandskatalog / competence-objected subject catalog
IMPP: Institut für Medizinische und Pharmazeutische Prüfungsfragen / Central German Institute for Medical and Pharmaceutical Examinations
SD: Standard Deviation
